# Vascular restenosis in coronary artery bypass grafting might be associated with VEGF-C/VEGFR-3 signaling pathway

**DOI:** 10.1007/s00380-018-1158-9

**Published:** 2018-03-20

**Authors:** Zuzanna Podemska-Jedrzejczak, Agnieszka Malinska, Patrycja Sujka-Kordowska, Michal Nowicki, Mateusz Puslecki, Marek Jemielity, Bartlomiej Perek

**Affiliations:** 1 Department of Histology and Embryology, Poznan University of Medical Sciences, Swiecickiego 6 St, 60-781 Poznan, Poland; 2 Department of Anatomy and Histology, University of Zielona Gora, Zyty 28 St, 65-046 Zielona Gora, Poland; 3 Department of Cardiac Surgery and Transplantology, Poznan University of Medical Sciences, Dluga 1/2 St, 60-101 Poznan, Poland

**Keywords:** Vascular endothelial growth factor, Caveolins, Graft patency, Coronary artery bypass grafting, Prognosis

## Abstract

The vascular endothelial growth factor (VEGF) family of peptides and caveolins (CAVs) are reported to contribute, in early graft failure in patients, a coronary artery bypass grafting (CABG). To investigate the possible association of ultimate luminal occlusion to VEGFs and CAVs expression, a functional analysis (based on the molecular biology, bioinformatics, histology, and clinical studies) was performed. Twenty-four hundred and sixty-eight CABG patients diagnosed with multivessel stable coronary artery disease (CAD) were enrolled into prospective study and assigned to two subgroups: double- and triple-vessel CAD subjects. Distal parts of all the harvested saphenous vein (SV) and internal thoracic artery (ITA) segments were used for further tests. ITA graft failure did not differ between double-vessel and triple-vessel CAD patients. The number of SV occlusions was significantly higher in triple-vessel CAD subjects. The microarray analysis performed on SV and ITA samples obtained exclusively from triple-vessel CAD patients who developed early graft occlusion revealed 383 genes with increased and 301 genes with decreased expression in ITA samples as compared to SV grafts. This was followed by functional analysis of ‘blood vessel development’ group of genes. Average *VEGF-C* expression in ITA grafts was higher than in corresponding SV grafts; *FLT4* expression was significantly higher in SV than in ITA transplants. VEGFR-3 and CAV3 expression demonstrated immunohistochemically in SMCs of the tunica media of SV grafts predicted their early restenosis in triple-vessel CAD patients. CAV2 protein expression in SMCs of ITA grafts indicated the risk of early graft failure both in double-vessel and triple-vessel CAD subjects.

## Introduction

Vascular restenosis is a common adverse event following coronary artery bypass grafting (CABG) [1]. One of the most important factors leading to graft occlusion is neointimal hyperplasia following both systemic (i.e., metabolic diseases, atherosclerosis, and hypertension) and local (i.e., adaptation to left-sided circulation) disorders [2, 3]. It has been observed that a majority of vascular grafts (both venous and arterial) lose their intimal endothelium soon after transposition into the coronary circulation [4, 5]. In the subsequent days, the endothelium undergoes reconstruction due to circulating endothelial cells (ECs) and tissue growth factors, including the vascular endothelial growth factor (VEGF) family of peptides [5, 6].

In line with the above process, the significance of VEGFs in vascular graft patency rate should be considered from two different points of view. Early VEGFs activity, defined by ECs restoration, must be regarded as the desirable (favorable) factor. On the other hand, the long-term VEGF activity in vascular grafts might result in smooth muscle cells (SMCs) proliferation and/or neointimal formation. In addition, this can promote the development of graft failure and occlusion. Monitoring the activity of the VEGF peptides after CABG is not feasible, because the graft is not accessible. Evaluating plasma VEGFs concentrations does not provide information that correlates with the actual condition in the vascular graft [7, 8]. Therefore, it appears to be especially important to define the pre-CABG tissue status of vascular grafts to determine the most accurate pattern of protein expression, which can then be used to predict its future condition. Several prospective studies have been performed so far. They focused on the determination of ECs markers [9, 10], immune cells infiltration [11], or caveolin (CAV) expression [12, 13]. The latter deserve special attention. CAVs and VEGFs appear to be complementary in predicting graft patency [14, 15]. This means that the VEGFs expression and function cannot be evaluated without a careful interpretation of the CAVs presence in particular parts of the vascular wall.

So far, there are no complex studies involving the use of microarray tools in determining the possible role of ‘blood vessel development’ group of genes in the long-term patency rate of vascular grafts. The majority of clinical and experimental trials have considered the potential roles of different angiogenic factors selected by research groups based on careful literature studies [16, 17]. In line with the information presented above, the present report, based on a combination of molecular, bioinformatics, histological, and clinical studies, seeks to define the actual significance of the VEGF and CAV families of peptides in vascular grafts restenosis.

## Materials and methods

### Study population

Twenty-four hundred and sixty-eight patients diagnosed with multivessel stable coronary artery disease (CAD), who underwent elective on-pump CABG with the use of at least one venous aortocoronary bypass graft between September 2008 and April 2014 in Department of Cardiac Surgery, Poznan University of Medical Sciences, were enrolled into this study. After approval of the study protocol by the Ethics Committee (Approval No. 1201/08), the patients were assessed for eligibility criteria. As many as 945 subjects were not admitted to the study as at least one of the following criteria was present: (1) harvesting of vessel graft other than ITA or SV, (2) class IV heart failure according to the NYHA, (3) cardiogenic shock, (4) single-vessel coronary disease, (5) diabetes, (6) coexistence of chronic renal failure or chronic renal disease, or (7) heart valve disease. A total of 1318 patients provided written consent and were allocated into two subgroups: the double-vessel CAD group (*n* = 375) and triple-vessel CAD group (*n* = 943). Three cardiac surgeons (with at least 10-year experience in CABG) implanted 3579 grafts (1318 ITA and 2261 SV) in these groups of patients.

**Fig. 1:**
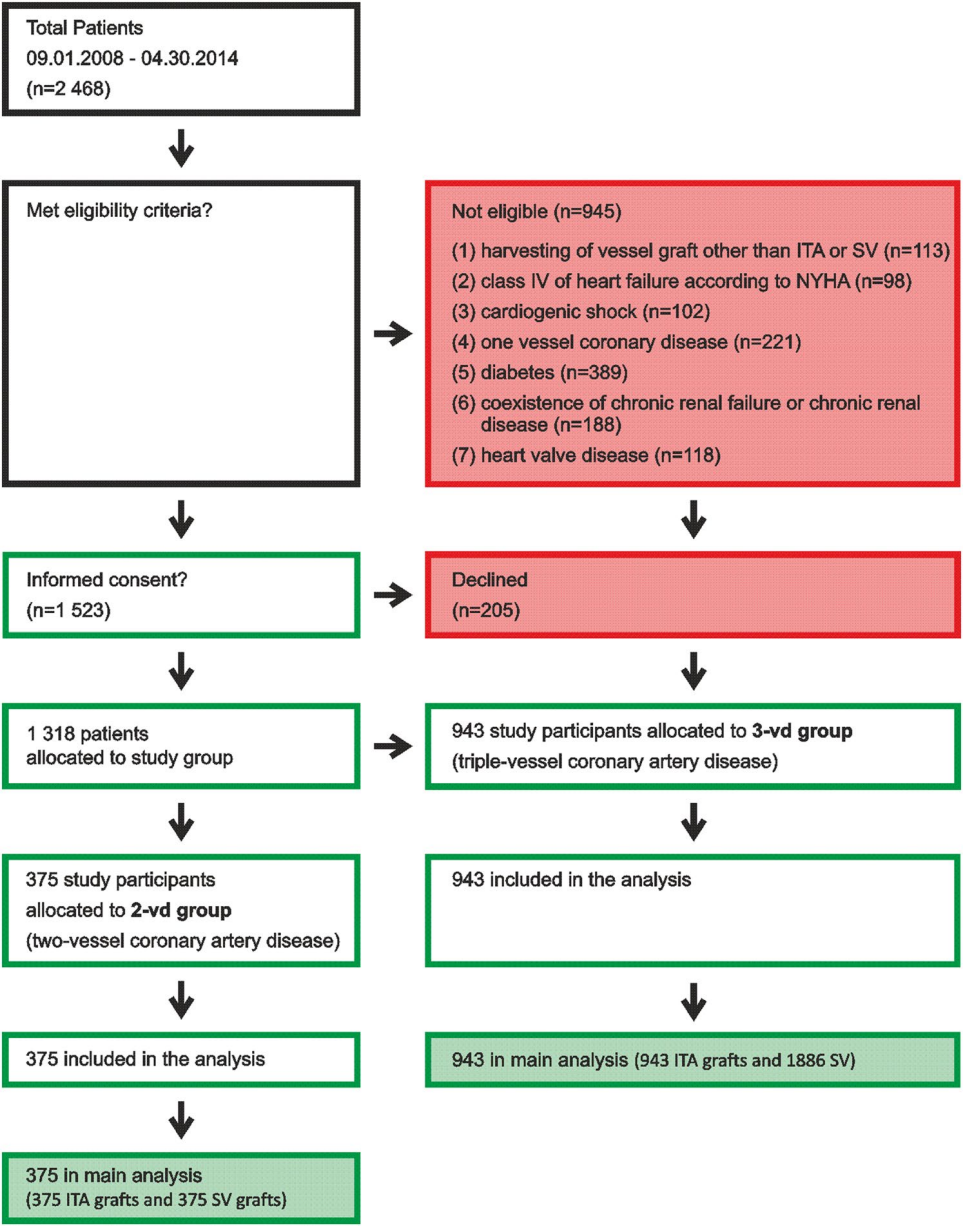
Diagrammatic representation of the sample size. *ITA* internal thoracic artery, *SV* saphenous vein

A diagrammatic representation of the sample size is presented in Fig. 1. Detailed preoperative demographics and clinical history data are summarized in Table 1.

**Table 1:** Patients’ demographics and selected laboratory and clinical data

Parameter	2-vd group (*n* = 375)	3-vd group (*n* = 943)	*p* value
Age (mean ± SD; range) (years)	64.2 ± 9.2; 48.2–73.1	68.1 ± 10.8; 49.5–79.9	ns
Gender (*M*/*F*)	270/105	634/309	ns
Arterial hypertension, *n* (%)	282 (75)	679 (72)	ns
Hyperlipidemia, *n* (%)	201 (54)	558 (59)	ns
Lipoprotein A (mg/dL)	30.7 ± 5.9	34.2 ± 6.1	ns
Family history of ischemic heart disease, *n* (%)	118 (32)	364 (39)	ns
Active smoking, *n* (%)	122 (32)	362 (39)	ns
History of smoking, *n* (%)	135 (36)	390 (42)	ns
Target vessels for implantation, *n* (%)			
D	13 (3)	194 (21)	0.008
LAD	356 (95)	915 (97)	ns
LCx	172 (46)	775 (82)	0.021
LM	180 (48)	688 (73)	0.033
PDA	8 (2)	66 (7)	ns
RCA	21 (6)	191 (20)	0.018

*2-vd* double-vessel coronary disease, *3-vd* triple-vessel coronary disease, *ns* not significant, *active smoking* considered if smoking cessation up to 1 year prior to surgery, *history of smoking* if smoking cessation between 10 and 1 year ago, *D* diagonal coronary artery, *LAD* left anterior descending coronary artery, *LCx* left circumflex coronary artery, *LM* left marginal coronary artery, *PDA* posterior descending coronary artery, *RCA* right coronary artery

### Operation procedure and sample collection

Cardiac muscle revascularization in double-vessel CAD patients involved the use of one ITA and one SV graft. On the other hand, triple-vessel CAD treatment usually included one ITA and two SV grafts.

In the majority of patients, a left ITA was used to bypass a left anterior descending coronary artery (LAD) and a right ITA was connected to the right coronary artery (RCA). The rest of the target coronary arteries (diagonal, left circumflex, obtuse marginal, and posterior descending) were supplied by SV grafts.

None of the patients received topical antibiotics at the time of surgical wound closure. The wounds were painted with povidone-iodine ointment and covered with a sterile dressing. Per study protocol, blood chemistry complete blood counts, and serum creatinine were obtained preoperatively and every alternate day. The study subjects received intravenous ceftazidime pentahydrate and intravenous amikacin sulphate via injection at the time of anesthesia induction; a second dose was given if the duration of surgery exceeded 5 h.

All surgeries were performed from median sternotomy. SV grafts were obtained through a full-length thigh incision over its course [18]. Key points of the procedure included minimal manipulation of the graft (“no-touch” technique) using low-intensity electrocautery and the control of the branches with stainless-steel vascular clips. In all the cases, distal part of harvested SV segment (at least 15–20 mm in length) was saved for subsequent laboratory studies.

ITA conduits were harvested as pedicles, together with satellite veins and endothoracic fascia. This was followed by opening the pericardium and performing an aortic cannulation. The distal end of the ITA segment was divided at the level of its bifurcation. After heparinization, ITA conduits were clipped distally, injected with 10 mL of a papaverine solution (1 mg/mL), and allowed to pharmacologically dilate. Immediately before ITA placement into the coronary circulation, a 10-mm segment of the conduit was harvested for further molecular and immunohistochemical tests.

### Follow-up

12 months after CABG, all the patients were evaluated via 64-slice multidetector computed tomography (CT). SV grafts with no evidence of luminal stenosis were classified as “patent”. Diseased conduits were classified as “single” when there was a single, local lesion; “multiply” when there was more than one lesion, but it was still not totally occluded; and “occluded” when no lumen was found in the CT examination [19].

### RNA isolation

RNA isolation from homogenized parts SV and ITA samples (including all the vessel layers) was carried out with the use of TRI reagent (T9424; Sigma-Aldrich, Poznan, Poland). Isolated RNA was subsequently purified on columns (RNeasy mini kit; Qiagen, Hilden, Germany) and the quantity of the total RNA was estimated spectrophotometrically (260 nm). RNA purity was determined based on the 260/280 nm absorption ratio (> 1.8) (NanoDrop spectrophotometer; Thermo Scientific, Waltham, MA, USA). Its integrity and quality were examined using a Bioanalyzer 2100 (Agilent Technologies, Inc., Santa Clara, CA, USA). Evaluated RNA Integrity Numbers (RINs) were between 8.5 and 10 with an average of 9.2 ± 0.7. Finally, the RNA concentration in each sample was diluted to 100 ng/μL and kept at − 80 °C until subsequent tests.

### Microarray analysis

Initially, isolated RNA (100 ng) was mixed with 1.5 μL of Poly-A RNA control solution and subjected to reverse transcription. The obtained cDNA was used for in vitro transcription to prepare antisense RNA (aRNA) by incubation samples at 40 °C for 16 h. It was then used for the second round of sense cDNA synthesis using a WT Expression kit (Ambion, Austin, TX, USA). All subsequent steps were performed in line with the recommendations provided with the Affymetrix microarray system (Affymetrix, Santa Clara, CA, USA). The obtained cDNA was used for biotin labeling and fragmentation using the Affymetrix GeneChip^®^ WT Terminal Labeling and Hybridization system. Biotin-labeled fragments of cDNA (5.5 μg) were hybridized to Affymetrix Human Gene HG-U219 Array Strips including unique oligonucleotide probes for a complex analysis of > 36,000 genes. Following hybridization, each array strip was washed and stained using the Fluidics Station of the GeneAtlas system (Affymetrix). The array strips were subsequently scanned using the Imaging Station of the GeneAtlas system and the preliminary analysis was performed with the use of Affymetrix GeneAtlas^™^ Operating Software. Fluorescence intensity was numerically converted to specific files containing output data for each microarray block.

All presented analyses and graphs were constructed using Bioconductor and the R programming language. The statistical significance of the analyzed genes was examined using moderated *t* statistics from the empirical Bayes method. The obtained *p* values were corrected for multiple comparisons using Benjamini and Hochberg’s false discovery rate [20]. The selection of genes with a significant change in expression was based on a *p* value < 0.05 and a ≥ twofold change in expression.

### Functional analysis by GeneAnswers and DAVID

To extend gene expression profiling, by creating inferred networks that might provide an interpretable structure of the gene list and visualize gene interactions, two novel gene-concept network analysis tools available as an open source packages were involved. The first one—GeneAnswers was employed not to create a gene-concept network, but it was also used to build protein–protein interactions networks. The second web-accessible program—DAVID (Database for Annotation, Visualization and Integrated Discovery) provided a comprehensive set of functional annotation tools to understand biological meaning expressed behind the list of genes (https://david.ncifcrf.gov/) [21].

Obtained lists of differentially expressed genes were combined as tables and subjected to a Gene Set Enrichment Analysis (GSES). According to assumptions used in the present study, a ‘blood vessel development’ group of genes was taken into account as a way to assess complex bioinformatics using the Kyoto Encyclopedia of Genes and Genomes (KEGG), Gene Ontology (GO), and BioCarta network databases. This was followed by calculation of enrichment score (ES), enabling standardization of studied group of genes according to their impact on the reconstruction of vascular walls leading to neointimal hyperplasia and loss of patency. Finally, string-db.org service devices were employed to visualize the functional protein association network.

### Validation via RT-qPCR

Reverse transcription, as performed in the present study, employed Avian Myeloblastosis Virus (AMV) Reverse Transcriptase (Promega Corp., Madison, WI, USA) with Oligo(dT) (PE Biosystems, Warrington, UK) as the primers at a temperature of 42 °C for 60 min (Thermocycler UNO II; Biometra, Goettingen, Germany). The primers used were designed by Primer 3 software (Whitehead Institute for Biomedical Research, Cambridge, UK) and subsequently purchased from the Laboratory of DNA Sequencing and Oligonucleotide Synthesis, Institute of Biochemistry and Biophysics, Polish Academy of Sciences, Warsaw, Poland.

For human VEGF-C, the primers used were as follows: forward, 5′-CTACAGATGTGGGGGTTGCT-3′ and reverse, 5′-CATCCAGCTCCTTGTTTGGT-3′. Human FLT4 primers were as follows: forward, 5′-AGAGACTTCCTGAGCTGTTTCC-3′ and reverse, 5′-AAGCACAAGTGGTTCTGGGTGC-3′. Primers efficiency was calculated using CFX Manager Software (Bio-Rad, Warsaw, Poland). *GAPDH* was used as a reference gene (the studied tissue was not exhibited to any experimental factors which could influence its expression). RNA samples were not treated with DNAse. The dynamic range over which a reaction is linear is extended to 6 log_10_ concentrations.

Reactions were carried out in a volume of 25 μL of reaction mixture consisting of 1 μL cDNA, 12.5 μL of polymerase enzyme mixture together with SYBR Green (Maxima SYBR Green/ROX qPCR Master Mix, ThermoFisher Scientific, USA, cat no. K0221), and 11.5 μL nuclease-free water. RT-qPCR was performed using Lightcycler 2.0 instrument (Roche Diagnostics Corp., Indianapolis, IN, USA) with software version 4.05. The real-time PCR program included a 10-min denaturation step to activate the TaqDNA polymerase, followed by a 3-step amplification program: denaturation at 95 °C for 10 s, annealing at 56 °C for 5 s, and extension at 72 °C for 10 s. The specificity of the reaction products was examined by determining the melting points (0.1 °C/s transition rate).

### Immunohistochemistry

The relevant segments of SVs and ITAs harvested from all the patients in the double-vessel (*n* = 375) and triple-vessel (*n* = 943) CAD groups, were immediately rinsed in 0.9% NaCl and fixed in Bouin’s solution for 24 h. They were subsequently dehydrated and then embedded in paraffin blocks following a routine procedure. Finally, they were cut into 3– 4-µm-thick sections on a semi-automatic rotary microtome (Leica RM 2145, Leica Microsystems, Nussloch, Germany). Each individual SV and ITA sample (taken from separate patients) was cut into approximately 10 paraffin sections. All of the immunohistochemical (IHC) analyses employed the StreptABComplex/HRP method modified by the use of biotinylated tyramine (Dako Catalyzed Signal Amplification System, Peroxidase, K1500, DakoCytomation A/S, Glostrup, Denmark). The endogenous peroxidase activity was blocked with 10% hydrogen peroxide. The indirect ABC IHC technique was performed in the following consecutive steps: (1) preincubation with the appropriate normal goat serum in phosphate-buffered saline (PBS) for 30 min at room temperature, (2) incubation with the specific primary antibody overnight at 4 °C in a hybridization chamber, (3) incubation with the secondary antibody for 60 min at room temperature, and, finally, (4) antigen–antibody complexes staining using 0.5% 3–3′ diaminobenzidine (DAB; Sigma Chemical Co., St. Louis, MO, USA).

The specific primary antibodies used in the study protocol together with the working solutions are summarized in Table 2. A semi-quantitative analysis of the IHC results was performed based on IRSs (immuno-reactive scores) according to Remmele and Stenger [22] providing a sufficient accuracy combined with high practicability [23]. For these scores, cytoplasmic protein expression was defined as negative (IRS 0–1), positive weak (IRS 2–3), positive moderate (IRS 4–6), or strong (IRS 8–12) (Table 3). The quantitative assessment of the studied proteins from ECs of tunica intima samples was performed by comparing the length of the immunopositive endothelium to the total length of the endothelium. The ratio of positive ECs was calculated with the use of AxioVision image processing software (Carl Zeiss MicroImaging GmbH, Göttingen, Germany) (Table 2). All tissue sections were analyzed under an AxioImager Z.1 light microscope and selected pictures were taken with attached AxioCam MRc5 digital camera (Carl Zeiss).

**Table 2:** Antibodies used in the studies

Antibody	Concentration	Code	Host	Manufacturer
Anti-VEGF(A)	1:100	M7273	Mouse-monoclonal	Dako Gdynia, Polska
Anti-VEGF(B)	1:300	27220002	Rabbit-polyclonal	Novus Biologicals, Littleton CO, USA
Anti-VEGF(C)	1:100	NBP1-18626	Mouse-monoclonal	
Anti-VEGFR1	1:400	NB100-527	Rabbit-polyclonal	
Anti-VEGFR2	1:400	NB100-627		
Anti-VEGFR3	1:200	NBP1-18651	Mouse-monoclonal	
Anti-CAV1	1:200	sc-894	Mouse-monoclonal	Santa Cruz Biotechnology Inc., Heidelberg, Germany
Anti-CAV2	1:800	sc-7942	Rabbit-polyclonal	
Anti-CAV3	1:200	sc-5310	Mouse-monoclonal	

*VEGF* vascular endothelial growth factor, *VEGFR* VEGF receptor, *CAV* caveolin

**Table 3:** Semi-quantitative analysis of the VEGF family of proteins and CAV tissue expression in internal thoracic artery and saphenous vein grafts from patients allocated to the double-vessel and triple-vessel CAD groups

Marker	2-vd group (*n* = 375)		3-vd group (*n* = 943)	
	ITA	SV	ITA	SV
VEGF(A)				
ECs^*^	96.2 ± 2.9	95.4 ± 3.4	98.1 ± 1.4	95.8 ± 3.3
SMCs^**^	7.3 ± 3.4	6.9 ± 3.7	7.5 ± 3.3	6.7 ± 2.9
VEGF(C)				
ECs	89.9 ± 5.1	–	93.9 ± 3.1	–
SMCs	5.8 ± 2.4	5.9 ± 3.9	5.5 ± 3.1	5.7 ± 2.8
VEGFR1				
ECs	97.2 ± 1.7	98.2 ± 1.4	97.7 ± 1.5	97.9 ± 1.5
VEGFR3				
ECs	–	85.1 ± 6.9	–	88.1 ± 9.1
SMCs	–	5.7 ± 4.1	–	5.8 ± 3.7
CAV1				
ECs	96.2 ± 2.9	95.4 ± 3.4	98.1 ± 1.4	95.8 ± 3.3
SMCs	8.2 ± 2.3	3.9 ± 1.7***	7.5 ± 3.6	3.6 ± 2.3***
SMCs	7.9 ± 2.7	–	8.3 ± 2.6	–
CAV3				
ECs	95.1 ± 2.8	–	93.2 ± 3.4	–
SMCs	8.1 ± 2.4	7.9 ± 2.8	8.5 ± 2.3	7.7 ± 2.1

*VEGF* vascular endothelial growth factor, *CAV* caveolin, *ITA* internal thoracic artery, *SV* saphenous vein, *CAD* coronary artery disease, *2-vd* two-vessel disease, *3-vd* triple-vessel disease, *VEGFR* VEGF receptor, “–” absence of the trait, *defined as the percentage of luminal endothelium, **according to IRS score, ***p = 0.001

An IHC analysis of protein expression in each vessel section was done within ten representative microscopic fields (200× magnification). All of the analyses were evaluated independently by two scientists on coded samples that included positive and negative controls. The negative controls consisted of specimens incubated with non-immune IgG1 (X-0931, Dako, Gdynia, Poland) and sections for which the primary or secondary antibody was omitted. All of the sections from blood vessels samples from an individual patient were processed in the same IHC experiment.

### Statistical analysis

All continuous variables are expressed as the mean ± SD. Initially, they were assessed for normality with the Shapiro–Wilk *W* test. When they showed a normal distribution, a subsequent analysis of variance (ANOVA) was employed, which was then followed by post hoc comparisons of means (Tukey honest significant difference test for an unequal samples sizes in a group—Spjotvoll/Stoline test). Non-parametric continuous variables were compared using the Kruskal–Wallis test. Pearson *χ*^2^ test was employed to analyze nominal data. The independent risk factors for graft occlusion were determined using Fisher’s exact test.

All of the statistical analyses were performed using the Statistica 12.0 PL software package (StatSoft, Poland). The results were reviewed every 3 months to enable the study to be terminated early if clear results emerged. Four interim analyses were performed during the trial. However, the results were not statistically significant until the final analysis was performed (after 24 months from the surgery). The interim analyses have not been considered in the sample-size calculation.

A value of *p* < 0.05 was considered to be statistically significant.

## Results

### 12-month follow-up

The study participants allocated to the double-vessel and triple-vessel CAD groups did not differ in demographics or preoperative clinical status (Table 1).

One year after CABG, 1715 SV grafts (81.8%) were classified as “patent”. One or more non-occlusive lesions were found in 211 conduits (10.1%). Finally, a complete occlusion was developed in 170 SV grafts (8.1%). In the same period of time, ITA grafts patency was as follows: “patent”—1401 grafts (94.5%), “single”—42 conduits (2.8%), “multiply”—12 grafts (0.8%), and “occluded”—28 (1.9%).

### Primary concept analysis

ITA graft failure did not differ between double-vessel and triple-vessel CAD patients. The number of recognized SV occlusions was, however, significantly higher in triple-vessel CAD study participants. For this reason, the initial screening with the use of microarray analysis was performed on SV (*n* = 18) and ITA (*n* = 21) samples obtained exclusively from triple-vessel CAD patients who developed graft occlusion within 12-month period after cardiac surgery.

**Fig. 2:**
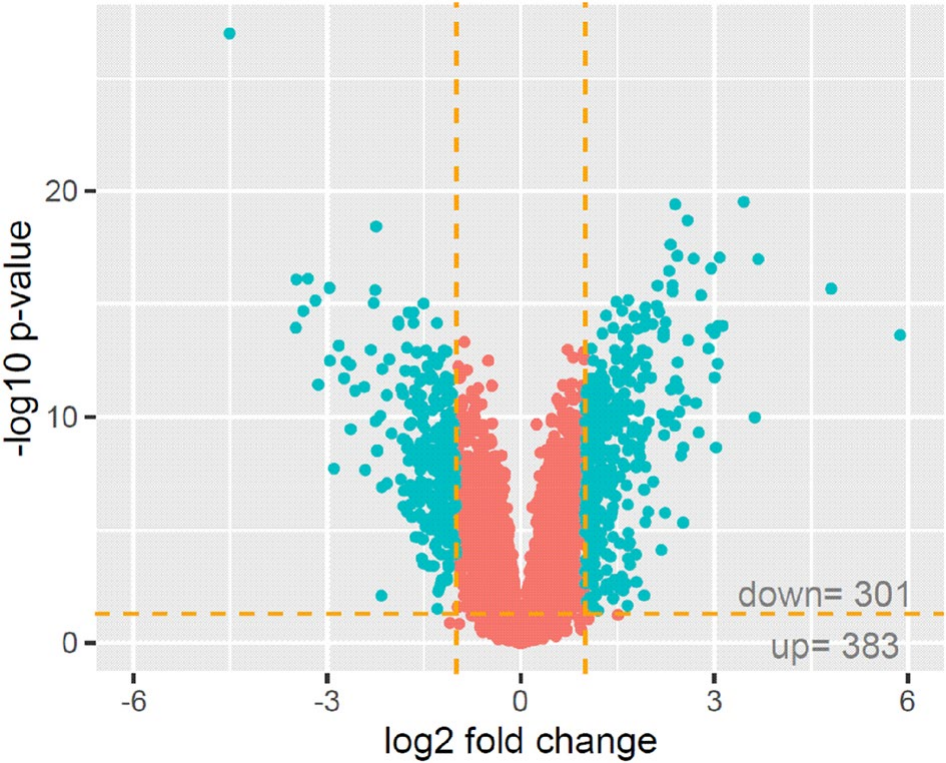
Volcano plot of the gene expression assay. By applying cut-off parameters (fold change ± 2; *p* < 0.05), Affymetrix Human Gene HG-U219 Array data revealed 383 genes with increased expression in internal thoracic artery samples compared to expression in saphenous vein (SV) samples and 301 genes for which expression was significantly lower than in SV samples. Aquamarine indicates genes that meet the cut-off parameters (fold change ± 2; *p* < 0.05); red indicates genes that did not meet the parameters

The mean expression value of each gene is presented as a volcano plot (Fig. 2). By applying the previously mentioned cut-off parameters (fold change ± 2; *p* < 0.05), Affymetrix Human Gene HG-U219 Array data revealed 383 genes with increased expression in ITA samples (as compared to SV samples) and 301 genes that showed significantly lower expression as compared to SV.

**Fig. 3:**
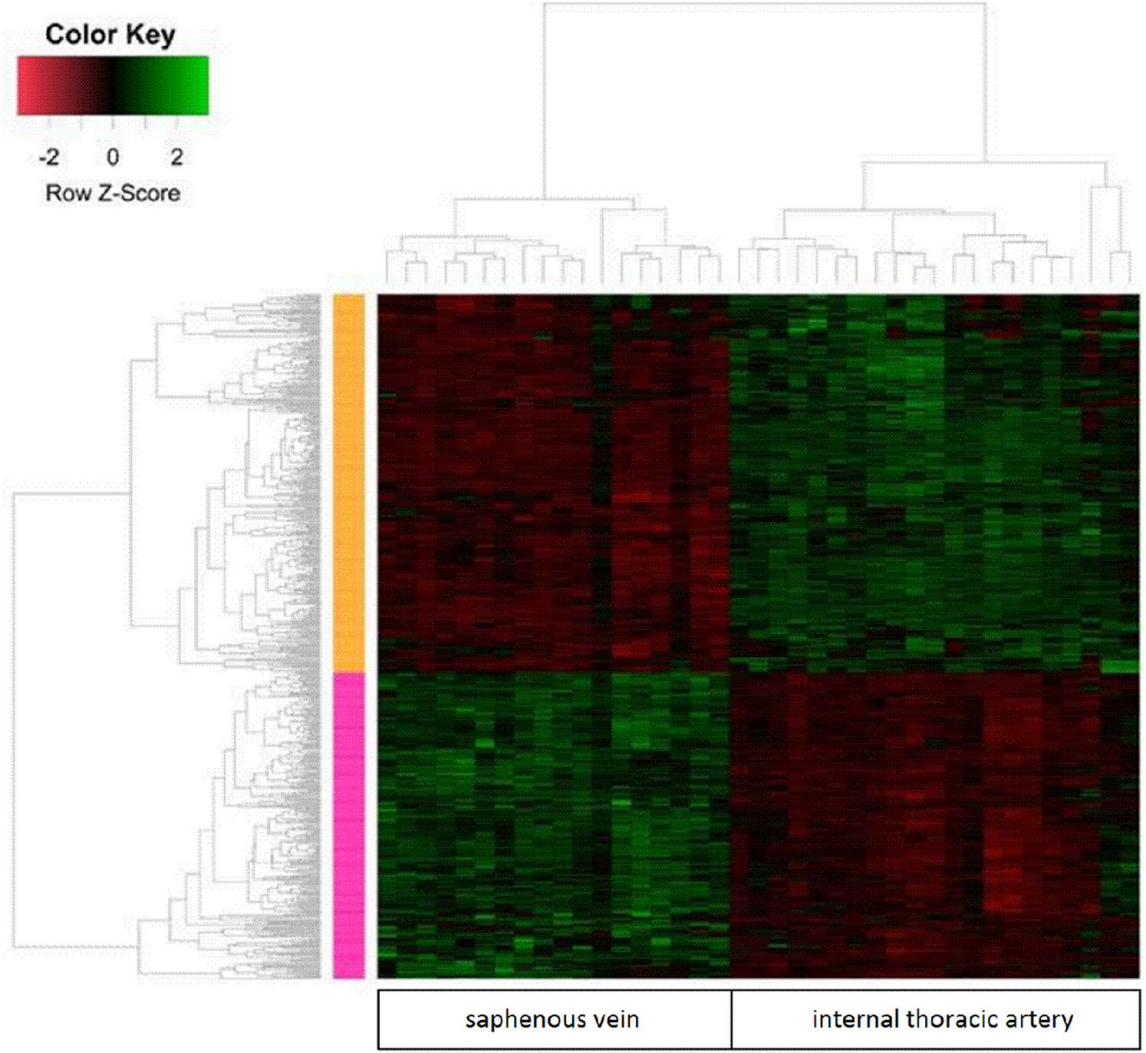
Heat map analysis of microarray data showing gene expression in vascular grafts. Heat map along with a functional clustering analysis related to genes (rows) and attempts (columns). Red and green show, respectively, reductions or increases in the expression of a given gene

**Fig. 4:**
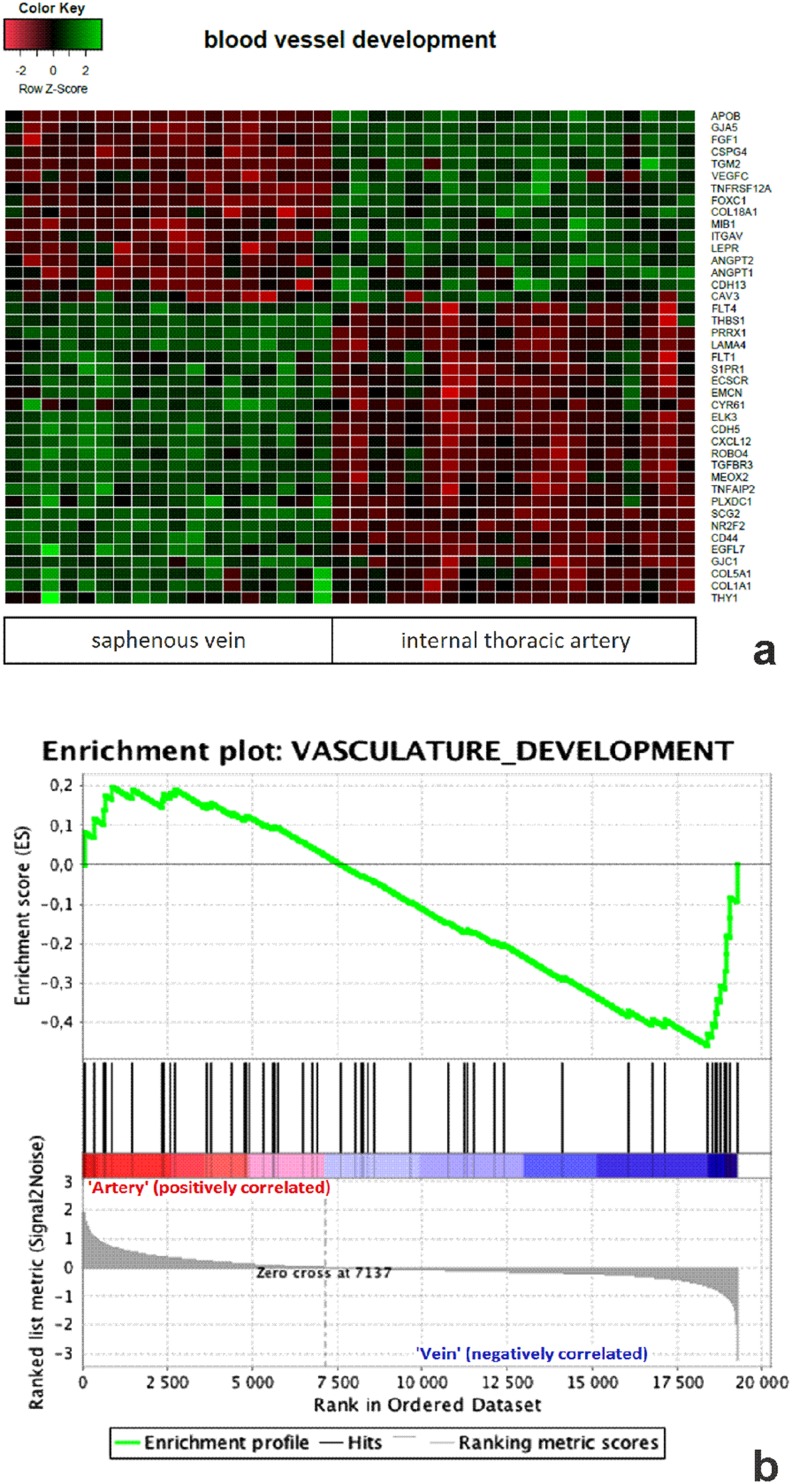
Gene mapping enabled the identification of the specific ontology group representing blood vessel development genes. Red and green show, respectively, reductions or increases in the expression of a given gene, which are listed in rows. Attempts are presented in columns. **a** Characteristics of individual genes within the ontology group and **b** results of the Gene Set Enrichment Analysis (GSEA)

The microarray analysis was followed by an analysis using a hierarchical clustering algorithm (Fig. 3). The results of this analysis, presented as a heat map, enabled the determination of stand-off values for some samples, which were excluded from further studies (two ITA specimens). In line with the above results, a functional analysis of the ‘blood vessel development’ group of genes was performed on 18 SV and 19 ITA samples (Fig. 4a). This type of clustering confirmed an increased expression of 16 genes and a decreased expression of 25 genes in ITA grafts compared to expression in SV transplants. In addition, a GSES analysis performed within the ‘blood vessel development’ ontology group showed results in parallel with the information presented above (Fig. 4b).

### Angiogenesis and graft failure

**Fig. 5:**
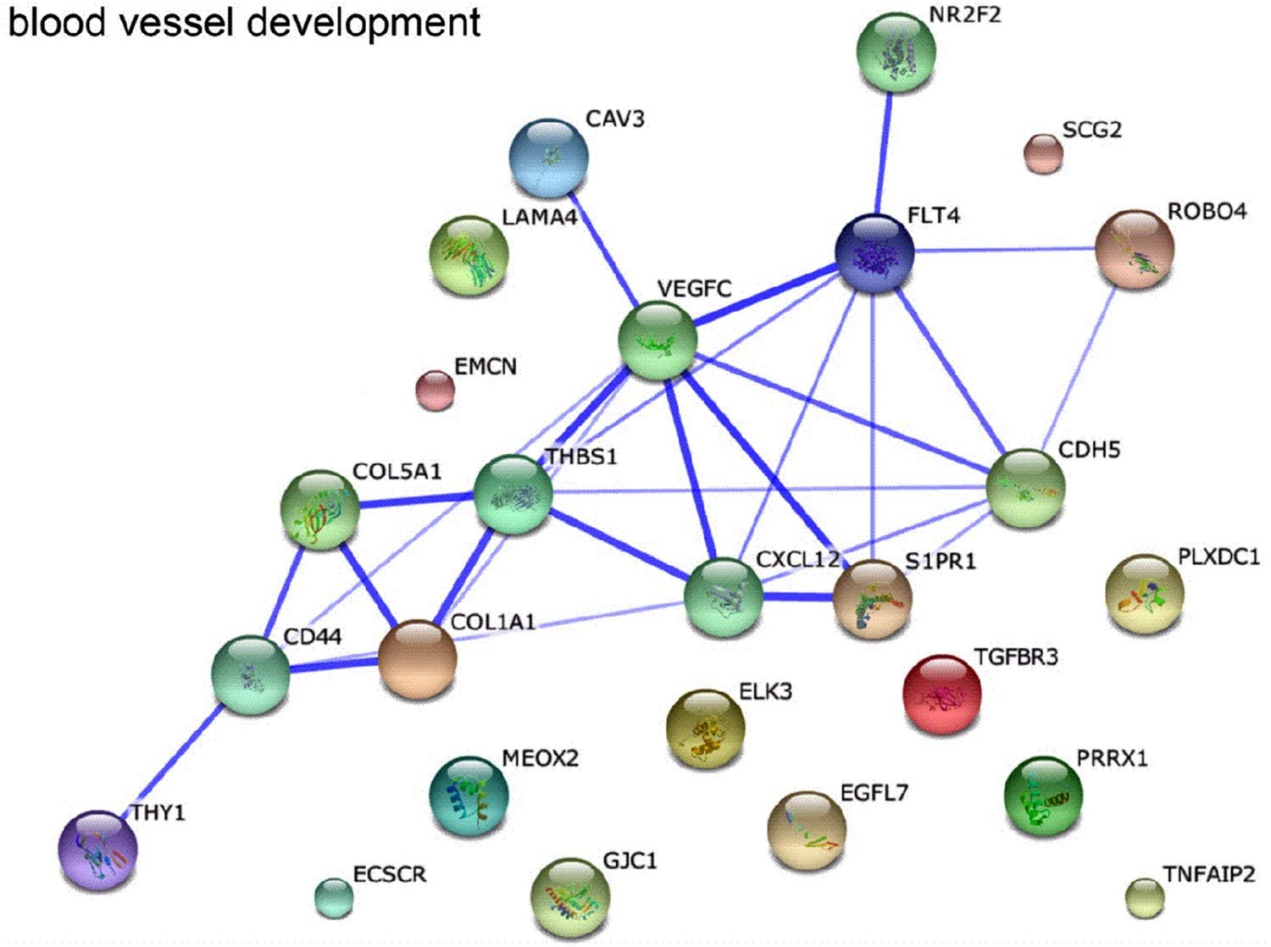
Relationship between individual genes composing the ‘blood vessel development’ ontology group. Diagram is based on searches of scientific article databases, experimental data, and data concerning gene expression and gene location. The thickness of the lines in the graph reflects the degree of gene co-expression as presented in the summaries of scientific publications

Advanced functional analyses performed with the use of DAVID and string-db.org tools made it possible to describe the most likely pattern of interactions present within ‘blood vessel development’ group of gene transcripts in ITA and SV transplants from triple-vessel CAD patients who developed graft failure within 12-month period after surgery (Fig. 5). It was found out that *VEGF-C* and *FLT4* are located in the main nodes of binding network illustrating the most likely pattern of interactions in graft failure development in these patients. What is more, triple-vessel CAD patients revealed significantly higher *VEGF-C* expression in ITA transplants and higher *FLT4* expression in SV grafts. For this reason, a subsequent analysis involved quantitative RT-qPCR measurements of *VEGF-C* and *FLT4* expression rates. The average *VEGF-C* expression rate in ITA grafts of triple-vessel CAD patients was 2.83 ± 0.4 higher than in corresponding SV grafts. On the other hand, *FLT4* expression was significantly higher in SV (3.22 ± 0.3) than in ITA transplants. It must be emphasized that the DAVID analysis only pointed the further research into the potential VEGF-C- and VEGFR-3-binding pathway (within ITA and SV grafts, respectively) which has been reported in subsequent parts of the present work.

### VEGF isoforms expression in vascular grafts tissues

Immunohistochemical studies were performed on all the tissue samples obtained from double-vessel (*n* = 375) and triple-vessel CAD patients (*n* = 943).

There was no overall difference in general IHC staining of VEGF-A in ITA and SV grafts in both study subgroups (Table 3). VEGF-A was present in SMCs of the tunica media and luminal endothelium. It was also present within small vasa vasorum of the adventitia.

In SV and ITA graft sections, the positive expression of VEGF-B was not identified.

**Fig. 6:**
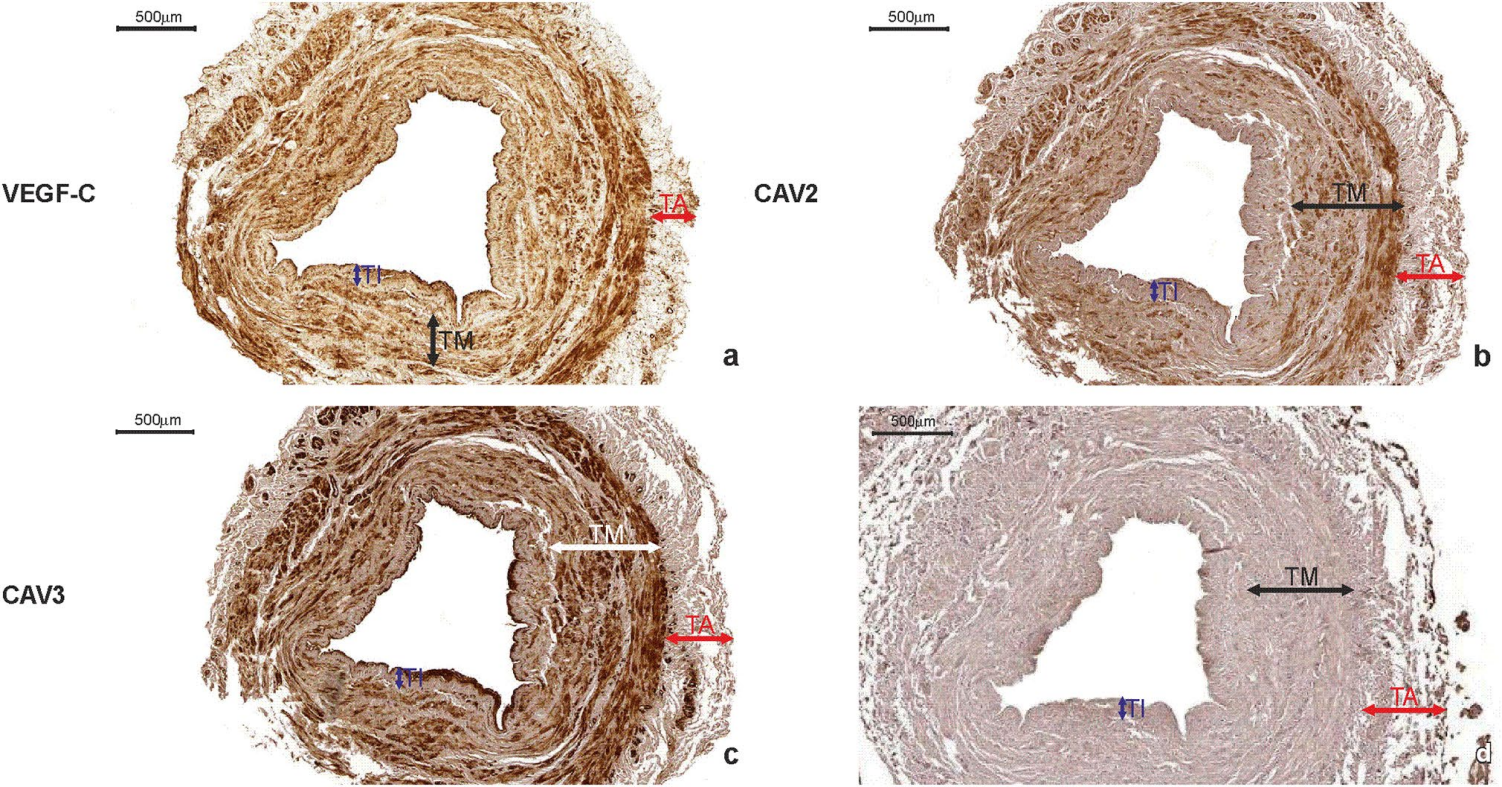
Internal thoracic artery wall harvested from a patient diagnosed with double-vessel CAD showing immunohistochemical staining. Immunohistochemical staining of VEGF-C (**a**), CAV2 (**b**) and CAV3 (**c**) in an internal thoracic artery obtained from a 67-year-old patient who developed the early graft restenosis within 7 months after coronary artery bypass grafting. VEGF-C is expressed in smooth muscle cells of the tunica media (TM) and endothelial cells present in the tunica intima (TI). CAV2 is present exclusively in smooth muscle cells. Strong expression of CAV3 is observed in smooth muscle cells and in endothelial cells. **d** Negative control. *TA* tunica adventitia

**Fig. 7:**
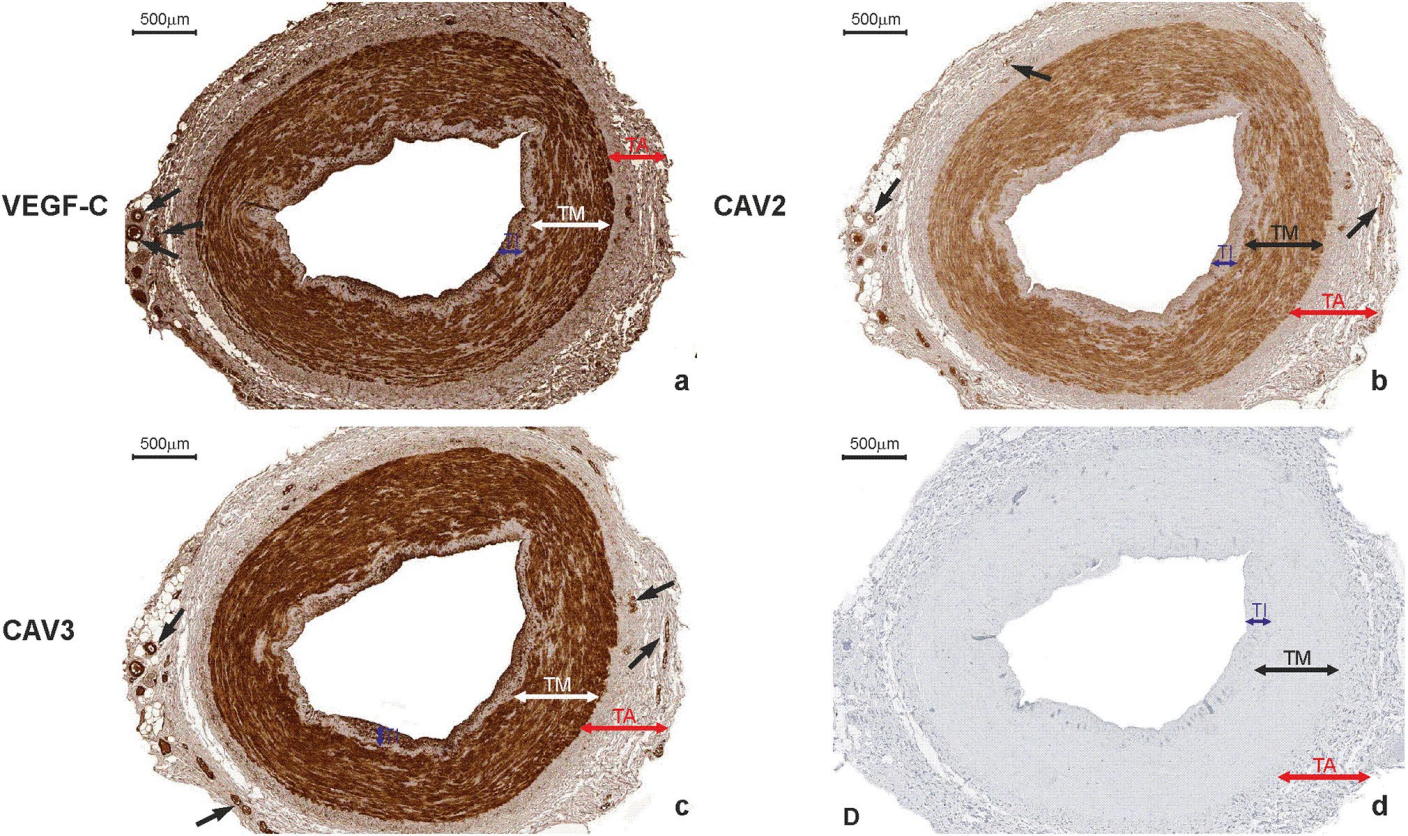
Immunohistochemistry of an internal thoracic artery wall harvested from a patient diagnosed with triple-vessel CAD. Immunohistochemical staining of VEGF-C (**a**), CAV2 (**b**), and CAV3 (**c**) in an internal thoracic artery graft harvested from a 71-year-old patient who developed early graft failure 11 months after coronary artery bypass grafting. Intensive VEGF-C and CAV3 expression is observed in the smooth muscle cells of the tunica media (TM) and in the endothelial cells of the tunica intima (TI). The presence of immunopositive expression of CAV2 (**c**) is limited to smooth muscle cells of the tunica media (TM). Expression of all analyzed proteins is present in the small blood vessels localized in the tunica adventitia (TA, arrows). **d** Negative control

**Fig. 8:**
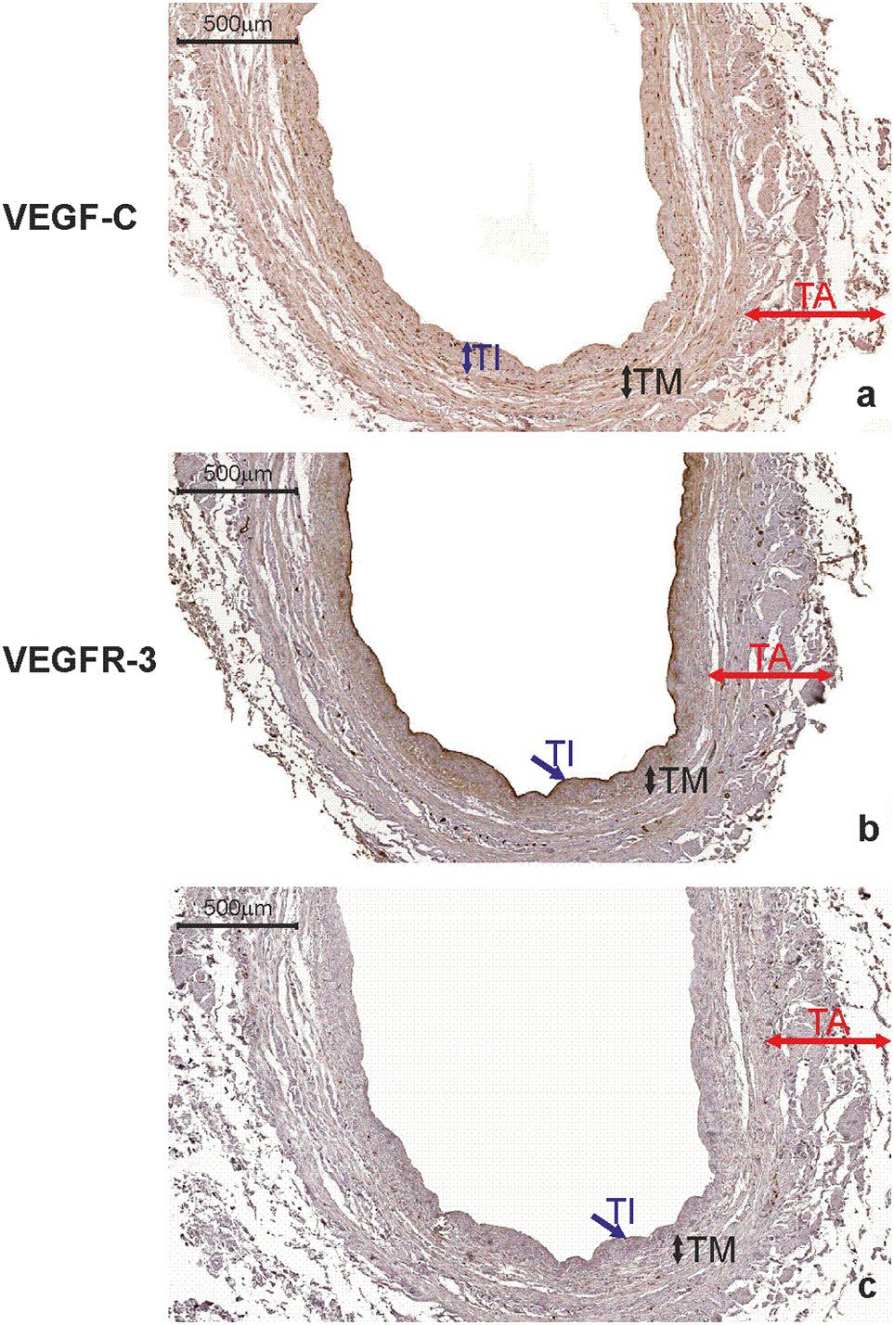
Saphenous vein wall harvested from a patient diagnosed with double-vessel CAD showing immunohistochemical staining. VEGF-C (**a**) and VEGFR-3 (**b**) reactivity in a graft wall obtained from a 67-year-old patient who developed the early graft restenosis within 7 months after coronary artery bypass grafting (the same patient as mentioned in Fig. 6). VEGF-C is localized exclusively within individual smooth muscle cells of the tunica media (TM). Cells with positive VEGFR-3 expression are situated in endothelial cells of the tunica intima (TI). **c** Negative control. *TA* tunica adventitia

**Fig. 9:**
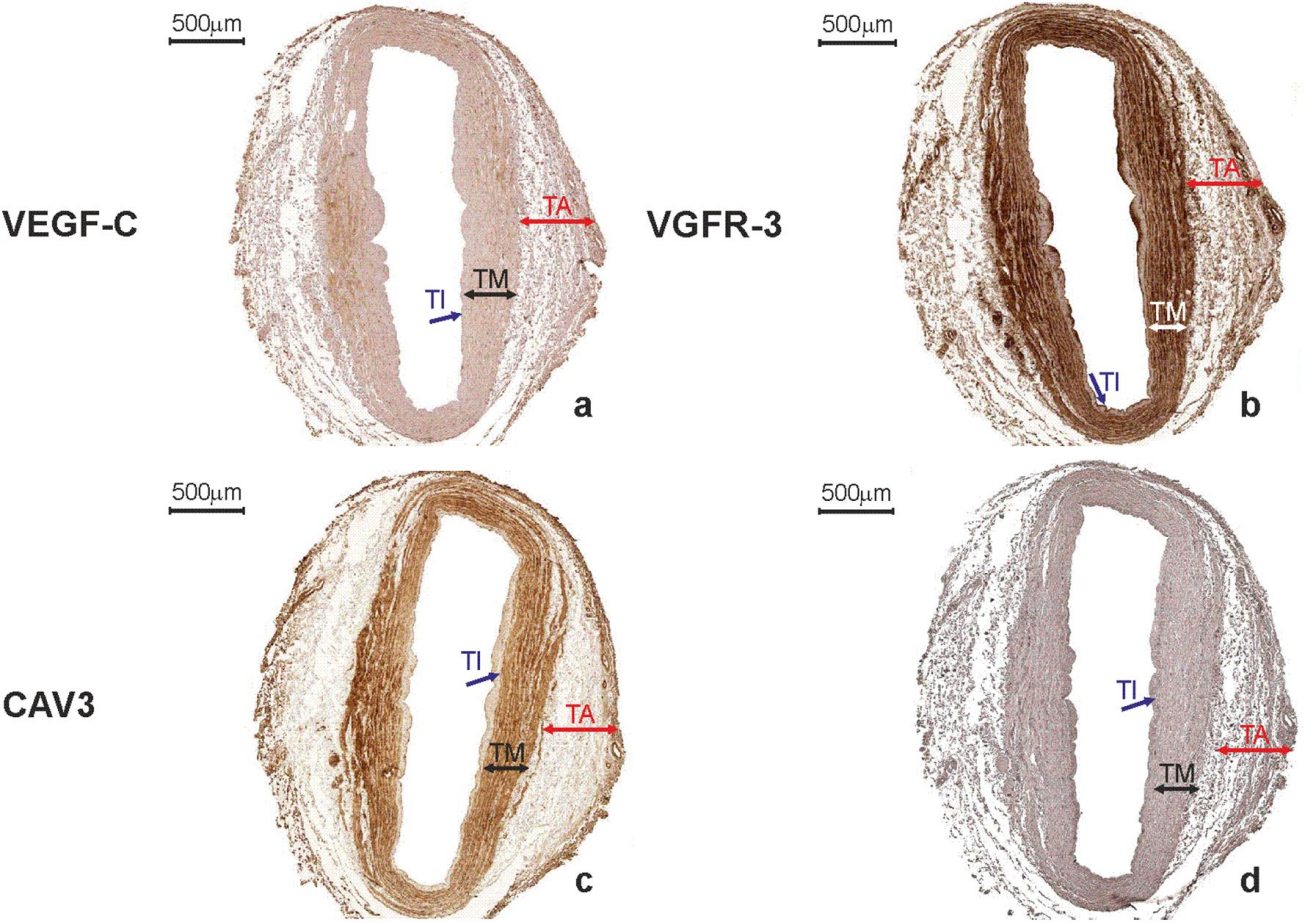
Immunohistochemical staining of a saphenous vein wall harvested from a patient diagnosed with triple-vessel CAD. Three serial histological sections of a graft wall obtained from a 71-year-old patient who developed early graft failure 11 months after coronary artery bypass grafting (the same patients as mentioned in Fig. 7). VEGF-C (**a**), VEGFR-3 (**b**), and CAV3 (**c**) shown to be expressed by immunohistochemical staining in smooth muscle cells of the tunica media (TM). In addition, VEGFR-3 is strongly present in endothelial cells of the tunica intima (TI). **d** Negative control. *TA* tunica adventitia

VEGF-C was expressed in 358 ITA grafts obtained from double-vessel CAD patients (95.5%) and 911 ITA transplants from triple-vessel CAD patients (96.6%). It was present within SMCs of the tunica media, ECs of the luminal endothelium, and individual vasa vasorum, as well as in the extracellular matrix (ECM) of the adventitia (Figs. 6a, 7a). There was no difference in VEGF-C expression between study participants who developed graft failure (subgroups: “single”, “multiply”, and “occluded”) and those who were classified as “patent” (Table 4). SV grafts obtained from double-vessel (Fig. 8a) and triple-vessel CAD patients did not expressed VEGF-C in the luminal endothelium (Fig. 9a). It was present in individual SV grafts within SMCs of the tunica media. Forty-two patients from the double-vessel CAD group (11.2%) and ninety-eight patients from the triple-vessel CAD group (10.4%) expressed VEGF-C in their SV grafts. The number of SV VEGF-C positive cases did not differ between the study subgroups.

**Table 4:** Association of ITA and SV graft occlusion with the expression of the VEGF family of proteins and caveolins in endothelial and smooth muscle cells of vascular transplants (*p* value) based on Fisher’s exact test

Marker	2-vd group (*n* = 23)		3-vd group (*n* = 139)	
	ITA	SV	ITA	SV
VEGFR-3				
Endothelium	–	ns	–	ns
Muscle	–	ns	–	**0.018**
CAV2				
Endothelium	–	–	–	–
Muscle	**< 0.0001**	–	**< 0.0001**	–
CAV3				
Endothelium	ns	–	ns	–
Muscle	ns	ns	ns	**0.026**

Bold value indicates a statistically significant difference with a *p*-value less than 0.05

*ITA* internal thoracic artery, *SV* saphenous vein, *2-vd* double-vessel disease, *3-vd* triple-vessel disease, *VEGF* vascular endothelial growth factor, *CAV* caveolin, *VEGFR* VEGF receptor, “–” absence of the trait

### VEGFR 1–3 expression in tissue samples

VEGFR-1 expression was constant across the tissues. It was present in intimal ECs of ITA and SV transplants. It was not observed in SMCs of the tunica media or in adventitial components. Interestingly, VEGFR-2 was not expressed at all in the studied specimens. Finally, VEGFR-3 staining differed between the tissues, with no staining in ITA grafts and various expression patterns in SV transplants. In general, VEGFR-3 was present in the luminal endothelium of SV grafts (Figs. 8b, 9b). For SMCs of the tunica media, the presence of VEGFR-3 was identified in 98 patients in the double-vessel CAD group (26.1%) and in 281 patients from the triple-vessel CAD group (29.8%). According to Fischer exact test, VEGFR-3 expression in SMCs of the tunica media was associated exclusively with graft failure in triple-vessel CAD patients (*p* = 0.018) (Fig. 9b). Double-vessel CAD patients showed no association between graft occlusion rates and VEGFR-3 expression (Table 4).

### VEGF family and CAVs co-expression

In general, there was no difference in CAV1 and CAV3 expressions in ITA transplants from the double-vessel (Fig. 6c) and triple-vessel CAD patients (Fig. 7c). These caveolins were present within SMCs of the tunica media and luminal ECs.

**Fig. 10:**
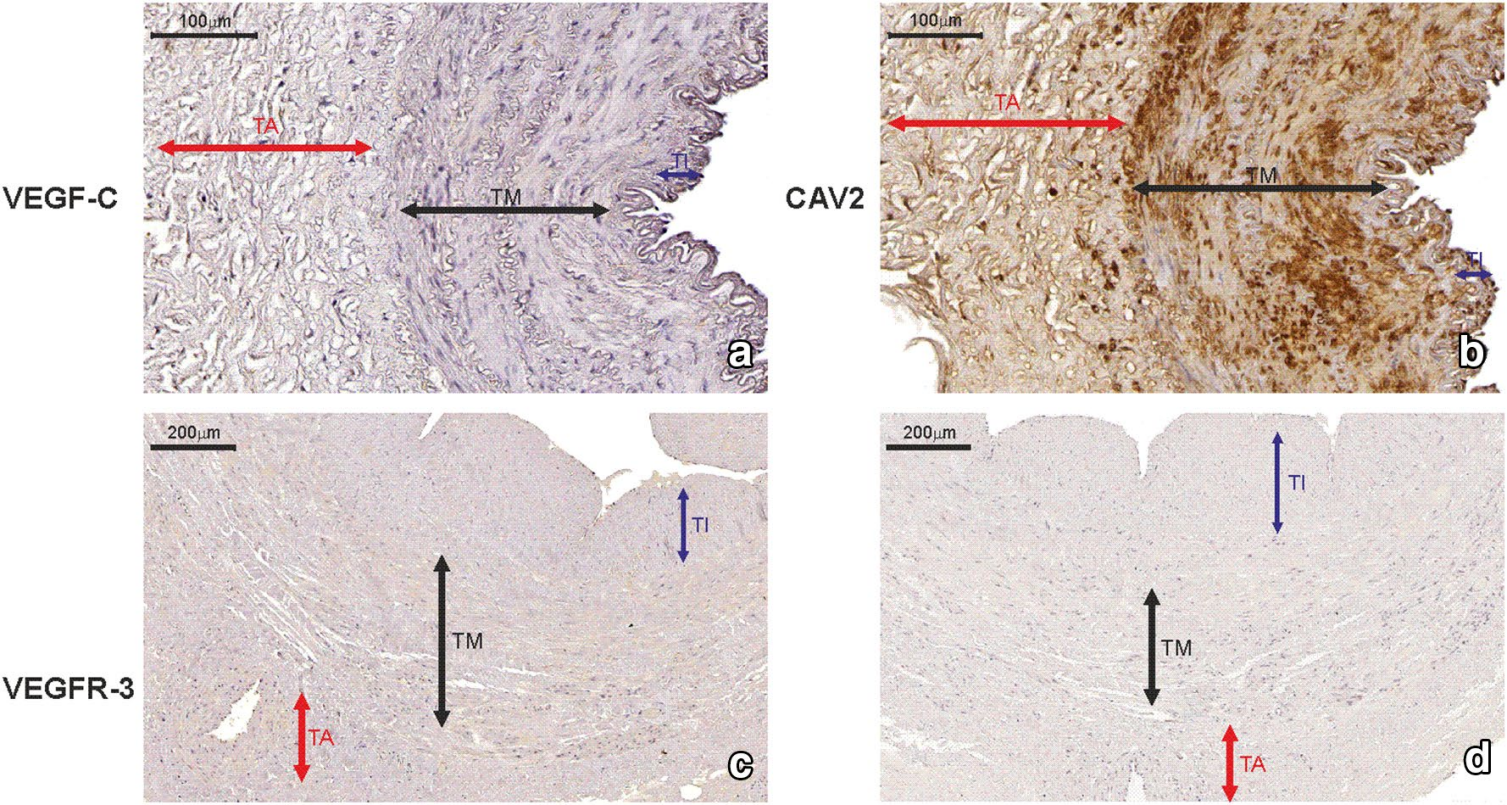
Immunohistochemical staining of an internal thoracic artery and saphenous vein wall harvested from a patient with no evidence of luminal stenosis 12 months after CABG. Immunohistochemical staining of VEGF-C (**a**) and CAV2 (**b**) in an internal thoracic artery (ITA) graft and VEGFR-3 (**c**) in saphenous vein (SV) transplant harvested from a 65-year-old patient diagnosed with triple-vessel CAD. Note that lack of CAV2 expression within smooth muscle cells (SMC) in ITA wall is accompanied by VEGF-C positive expression in the same area. In SV tissue sample harvested from the same patient, no VEGRR-3 expression was found. **d** Negative control. *TI* tunica intima, *TM* tunica media, *TA* tunica adventitia

Interestingly, CAV2 was expressed on the protein level within SMCs of ITA grafts in 7 study participants from the double-vessel CAD group (1.9%) (Fig. 6b) and 22 patients from the triple-vessel CAD group (2.3%) (Fig. 7b). Using Fischer’s exact test, it was determined that these patients developed ITA graft failure within the 12-month period of observation (*p* < 0.0001). What is more, in ITA, transplant from 358 (95.5%) patients from the double-vessel CAD group and 911 (96.6%) patients from the triple-vessel CAD group lack of CAV2 protein within SMCs was accompanied by VEGF-C positive expression (Fig. 10a, b). It should be emphasized that among these patients in 277 (74%) SV transplants harvested from the double-vessel CAD group and in 662 (70%) patients from the triple-vessel CAD, VEGFR-3 was not expressed within blood vessel wall (Fig. 10c). In this group of patients, no graft occlusion was observed. CAV1 expression within SV grafts did not differ between the studied subgroups. It was present within individual SMCs and the majority of ECs from vascular transplants. CAV2 was not expressed in these grafts. CAV3 immunoreactivity in SV samples was identified exclusively within SMCs of the tunica media (Fig. 9c). It was present in 87 subjects belonging to the double-vessel CAD group (23.2%) and 266 subjects in the triple-vessel CAD group (28.2%, the difference was not significant). What is more, CAV3 and VEGFR-3 were co-expressed in SV transplants from 82 patients from the double-vessel CAD group and 252 study patients from the triple-vessel CAD group. It should be emphasized that CAV3 expression in SMCs of the tunica media from SV grafts correlated to the early graft failure exclusively in triple-vessel CAD patients (*p* = 0.026). Detailed information is summarized in Tables 3 and 4.

## Discussion

The basic rationale for the present research was to optimize the clinical protocol of control examinations of the patients who have undergone CABG. We do realize that evaluation of all the CABG patients after surgery via CT is uneconomical. Therefore, an outpatient care system should be organized to provide a special care to those subjects who are at the highest risk of graft occlusion.

The best and most likely the only moment to determine the vascular conduit condition is the time of its implantation into the coronary circulation. Therefore, our principal aim was to characterize the activities of the VEGF family proteins in the context of vascular pathologies using bioinformatics tools.

The pleiotropic functions of VEGF proteins include, among others, autocrine and paracrine effects on surrounding tissues [24, 25]. For this reason, an estimation of VEGF’s significance in the patency rates after CABG cannot be considered exclusively in the context of the type of vascular graft (ITA or SV) transplanted to the coronary circulation. The determination of possible VEGFs’ associations with graft patency must be based on the type of preliminary diagnosed coronary artery disease (i.e., single-vessel, double-vessel, or triple-vessel disease). This is due to the fact that usually both an ITA and one or more SV grafts are transplanted to the coronary circulation in multivessel CAD. Therefore, the long-term functioning of SV grafts may be affected by the presence of an ITA graft in the coronary circulation. It is well known that ITA grafting of the left anterior descending coronary artery (LAD) is associated with less progression of native atherosclerotic disease within the proximal LAD compared to when SV graft is anastomosed to this artery [26, 27]. This means that the clinical status of CABG patients after surgery depends on the type, number, and position of grafts used in cardiac muscle revascularization [28].

In the present study, the study participants underwent left ITA to LAD and right ITA to RCA anastomoses. SV graft(s) were used as vascular bridges in diagonal, left circumflex, obtuse marginal, and posterior descending coronary arteries (Table 1). The percentage of graft failure in the double-vessel CAD group was significantly lower than in the triple-vessel CAD group. Interestingly, in the former group, the numbers of ITA and SV grafts occlusions were similar. In the latter group, however, despite no differences in demographics and clinical statuses of the study participants, the number of SV graft failures was significantly higher. This might indicate that the long-term condition of venous grafts might be dependent on the presence of arterial (ITA) transplants in the coronary circulation.

So far, there are a few studies discussing the possible significance of the VEGF family of proteins in determining patients’ statuses after CABG operations. A few reports indicate the possible prognostic role of VEGF plasma levels in predicting short-term patency rates of vascular grafts [7, 8]. There are not, however, publications illustrating the tissue expression of the VEGF family of proteins in vascular grafts used in CABG.

In the present study, ITA and SV grafts (obtained from the same patients) were analyzed to assess the presence of possible interactions within the ‘blood vessel development’ group of gene transcripts. It was determined that triple-vessel CAD patients who developed graft failure within a 12-month period after surgery (Fig. 6) revealed a significant relationship among VEGF-C, FLT4, and CAV3 gene transcripts. For this reason, a systemic molecular and immunohistochemical evaluation of the VEGF and CAV families of peptides was performed.

Based on the above analysis, it was determined that the presence of VEGFR-3 and CAV3 in SMCs of the tunica media seems to be a useful marker in predicting the early SV graft restenosis in triple-vessel CAD patients. CAV2 protein expression in SMCs of ITA grafts can indicate the risk of their early failure both in double-vessel and triple-vessel CAD patients. This suggests that the key factors in the pathogenesis of the early graft restenosis in CAD patients are SMCs.

Looking more closely at Fig. 5, one can also notice thrombospondin 1 (THBS1), sphingosine-1-phosphate receptor 1 (S1PR1, endothelial differentiation gene 1), and collagens I and V, which seem to be significantly involved in the pathogenesis of early graft restenosis. THBS1 and S1PR1 are VEGFs-dependent proteins [29, 31]. They affect ECs in an opposite manner. THBS1 is regarded as an inhibitory agent, while S1PR1 stimulates ECs migration, capillary-like network formation, and vascular maturation [30, 31]. This means that, at the level of ECs, VEGF-dependent processes that stimulate and inhibit cell proliferation might be mutually balanced.

VEGF-A and VEGFR-1 were expressed constitutionally across SV and ITA grafts. The former one was present within SMCs and ECs, while the latter was present exclusively in ECs. Such a pattern might reflect the vascular capacity for constant endothelial restoration (as mentioned in the “Introduction”).

Interestingly, the process of fibrosis (an essential element of graft restenosis) is inhibited by CAV1, which downregulates VEGFs signaling [32, 33]. As was described in our results, CAV1 expression did not differ between studied subgroups and grafts; however, it was first of all present in ECs. Only individual SMCs were CAV1-positive. Following this observation, collagens I and V (dependent on COL1A1 and COL5A1 expressions, as presented in Fig. 5) may simply not be able to appear in the tunica intima. What is more, ITA grafts were found to be VEGFR-3-negative. Therefore, the potent process of neointimal formation in these grafts (predicted by CAV2 expression in SMCs) is most likely VEGFs-independent and still requires an explanation.

In summary, VEGF and CAV families of proteins seem to be involved in the pathogenesis of the early graft restenosis in CABG patients. Development of graft failure is, however, dependent on the number and the type of blood vessels (ITA or SV) transplanted to coronary circulation. Prediction of the early graft failure in CABG patients is, to some extent, possible by employing evaluation of VEGFR-3, CAV2, and CAV3 protein expression in SMCs of examined tissues. VEGFR-3 and CAV3 predict the early failure in SV grafts in triple-vessel CAD patients, while CAV2 indicates the risk of ITA graft restenosis in both double-vessel and triple-vessel CAD patients. Still, we are not able to indicate a clear marker of the early SV graft failure in double-vessel CAD patients.
